# pH Landscapes in a Novel Five-Species Model of Early Dental Biofilm

**DOI:** 10.1371/journal.pone.0025299

**Published:** 2011-09-23

**Authors:** Sebastian Schlafer, Merete K. Raarup, Rikke L. Meyer, Duncan S. Sutherland, Irene Dige, Jens R. Nyengaard, Bente Nyvad

**Affiliations:** 1 iNANO The Interdisciplinary Nanoscience Center, Faculty of Science, Aarhus University, Aarhus, Denmark; 2 Department of Dental Pathology, Operative Dentistry and Endodontics, School of Dentistry, Faculty of Health Sciences, Aarhus University, Aarhus, Denmark; 3 Stereology and Electron Microscopy Research Laboratory, Centre for Stochastic Geometry and Advanced Bioimaging, Aarhus University, Aarhus, Denmark; 4 Microbiology, Department of Biological Sciences, Faculty of Science, Aarhus University, Aarhus, Denmark; University of Kansas Medical Center, United States of America

## Abstract

**Background:**

Despite continued preventive efforts, dental caries remains the most common disease of man. Organic acids produced by microorganisms in dental plaque play a crucial role for the development of carious lesions. During early stages of the pathogenetic process, repeated pH drops induce changes in microbial composition and favour the establishment of an increasingly acidogenic and aciduric microflora. The complex structure of dental biofilms, allowing for a multitude of different ecological environments in close proximity, remains largely unexplored. In this study, we designed a laboratory biofilm model that mimics the bacterial community present during early acidogenic stages of the caries process. We then performed a time-resolved microscopic analysis of the extracellular pH landscape at the interface between bacterial biofilm and underlying substrate.

**Methodology/Principal Findings:**

Strains of *Streptococcus oralis*, *Streptococcus sanguinis*, *Streptococcus mitis*, *Streptococcus downei* and Actinomyces naeslundii were employed in the model. Biofilms were grown in flow channels that allowed for direct microscopic analysis of the biofilms *in situ*. The architecture and composition of the biofilms were analysed using fluorescence *in situ* hybridization and confocal laser scanning microscopy. Both biofilm structure and composition were highly reproducible and showed similarity to *in-vivo*-grown dental plaque. We employed the pH-sensitive ratiometric probe C-SNARF-4 to perform real-time microscopic analyses of the biofilm pH in response to salivary solutions containing glucose. Anaerobic glycolysis in the model biofilms created a mildly acidic environment. Decrease in pH in different areas of the biofilms varied, and distinct extracellular pH-microenvironments were conserved over several hours.

**Conclusions/Significance:**

The designed biofilm model represents a promising tool to determine the effect of potential therapeutic agents on biofilm growth, composition and extracellular pH. Ratiometric pH analysis using C-SNARF-4 gives detailed insight into the pH landscape of living biofilms and contributes to our general understanding of metabolic processes in *in-vivo*-grown bacterial biofilms.

## Introduction

Laboratory models simulating the microbial and chemical conditions that lead to tooth decay have a long history. As early as in the late 19^th^ century, Magitot exposed extracted human teeth to bacteriological media and reported that the carious lesions produced *in vitro* were identical to those occurring in a patient's mouth [1]. Since that time, experimental setups for the growth of bacterial biofilms mimicking dental plaque have constantly evolved. The past three decades in particular have seen a wide range of sophisticated models that permit standardization and modification of a multitude of parameters including temperature, nutrient flow, pH and microbial composition (see [2] and [3] for reviews). Although laboratory models will always fail to recreate the complexity of the oral environment, they offer many advantages. They circumvent the ethical conflicts that arise in clinical studies and permit to model and analyse a variety of important *in-vivo*-processes in a highly reproducible fashion. Dental biofilm models have contributed much to elucidate the cariogenic potential of different microorganisms [4]–[7], the role of microbial interactions in biofilm development [8]–[11] and the effect and working mechanisms of caries-preventive agents [12]–[17].

In caries aetiology, plaque pH plays an essential role. Microorganisms produce various organic acids from fermentable dietary carbohydrates. This lowers the pH at the tooth-biofilm-interface to ‘critical values’ below 5.5 [18] and leads to progressive demineralization of the tooth. Among the organisms involved in cariogenesis, *Lactobacillus* spp. and especially mutans-group streptococci (MS) have gained much attention. Both groups of organisms are highly acidogenic and aciduric and have been proven to effectively induce caries in experimental animals [19]. Their predominance in advanced carious lesions, determined by both culture-based and DNA-based approaches [20]–[22], has led to considering them as primary aetiological agents. However, a number of investigations have shown that other organisms, in particular *Actinomyces naeslundii* and the mitis-group streptococci *Streptococcus oralis*, *Streptococcus mitis*, *Streptococcus gordonii* and *Streptococcus sanguinis*, also display acidogenic and aciduric properties and are capable of creating a cariogenic environment [23], [24]. These organisms are among the earliest colonizers of the tooth and represent the majority of isolates from early plaque [25]–[28]. Moreover, they still outnumber MS and *Lactobacillus* spp. in mature dental biofilms on clinically sound tooth surfaces [29], [30], but also on incipient carious lesions [31], [32]. In some cases, neither MS nor *Lactobacillus* spp. may be present during early stages of lesion formation [31], [33], [34]. These findings have questioned the unique role of MS in initiating demineralization and promoted a new extended ecological hypothesis for explaining the role of bacteria in caries [35]. According to this hypothesis, two major bacterial shifts occur in the course of the pathogenetic process: First the proportion of highly acidogenic *Actinomyces* and non-MS strains rises, leading to the so-called acidogenic stage. Then even more acidogenic and aciduric species, such as MS and *Lactobacillus* spp., become established in the biofilm [36], [37], and the so-called aciduric stage is reached. To study the caries process, several highly acidogenic laboratory biofilms have been developed [6], [13]. However, no model mimicking the microflora present during mildly acidogenic stages of the caries process exists, although the early microbial community is of particular interest for caries-preventive approaches.

Measuring pH at the interface between substrate surface and bacterial biofilm is a challenge for dental research. The structure of dental biofilms is complex and heterogeneous. Differences in bacterial composition and activity as well as limited diffusion through the extracellular matrix result in a multitude of chemically diverse microenvironments in close proximity. While early attempts to analyse plaque pH were limited to measurements in bulk fluid [38], [39], the development of microelectrodes that can be introduced into the biofilm demonstrated the presence of pH gradients between biofilm surface and bottom that persisted for several hours after carbohydrate exposure [40], [41]. Only few investigations have tried to directly visualize pH gradients in living biofilms using non-destructive microscopy techniques. While pH-sensitive fluorescent dyes can readily be applied to determine the pH in intra- and extracellular compartments, their use in bacterial biofilms is hampered by inhomogeneous probe penetration, compartmentalization and fading, all of which can cause erroneous results. Ratiometric fluorescent dyes that undergo a pH-dependent wavelength shift overcome these limitations. They have been used in *Pseudomonas aeruginosa* biofilms [42] and Antarctic endolithic biofilms [43], and can be employed to capture detailed, time-resolved images of the pH landscape in living biofilms.

The objective of the present study was to develop and validate a 5-species model biofilm that mimics the mildly acidogenic conditions of the bacterial community present in young dental plaque. Strains *S. oralis* SK248 (OMZ 607), *A. naeslundii* AK6, *S. mitis* SK24 (NCTC 8029), the MS *Streptococcus downei* HG594 and *S. sanguinis* SK150 (CCUG 25605) were inoculated sequentially into a flow cell system that permitted direct microscopic examination, and biofilms were grown for 26 h at 35°C. We used a combination of fluorescence *in situ* hybridization (FISH) and confocal laser scanning microscopy (CLSM) to analyse the bacterial composition and architecture of the biofilms. We then stimulated living biofilms with glucose solutions and used the ratiometric fluorescent dye C-SNARF-4 (Seminaphtorhodafluor-4F 5-(and-6) carboxylic acid) and CLSM to monitor the time- and space-resolved pH-response at the biofilm-substratum interface.

## Results

### Bacterial composition and architecture of the model biofilm

**Figure 1:**
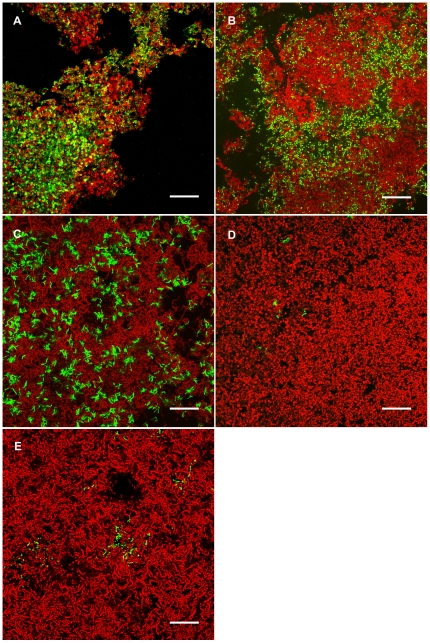
Biofilms hybridized with EUB338 and species-specific probes SMIT, SSAN, SORA2, SDOW or ANAES. EUB338 was labelled with Atto633 (red), species-specific probes were labelled with Cy3 (green). **A.** Biofilms were dominated by *S. mitis* SK24. **B.** Colonies of *S. sanguinis* SK150, shown in close colocalization with other streptococcal species. **C.** Cells of *A. naeslundii* AK6 formed branched spider-like colonies in basal layers of the biofilms. **D.** Small groups of *S. oralis* SK248 cells were scattered over the biofilm. **E.** Cells of *S. downei* HG594 appeared in particularly long chains intertwined with clusters of other streptococcal cells. Bars = 20 µm.

Dense biofilms had grown in the flow cells within 26 h after sequential inoculation of the five bacterial species. The surface topography was characterised by depressions and protuberances (Video S1), and biofilm thickness in the fields of view chosen for composition analysis varied between 7 and 68 µm, with an average thickness of 29 µm after FISH procedure. *S. mitis* SK24, which was the third strain to be inoculated, dominated the biofilm and accounted for 77.6% of the bacterial volume (Figure 1A). Although inoculated last, *S. sanguinis* SK150 represented 10.9% of the total biovolume and appeared as dense cell clusters, often interspersed with other streptococcal species (Figure 1B). *A. naeslundii* AK6 was inoculated second and accounted for 2.8% of the biovolume. The organism was rarely observed in upper layers of the biofilms, but represented 11.4% of the bacterial volume in the bottom layer where it formed branched, sometimes spider-like colonies (Figure 1C, Video S1).

In preliminary experiments, coaggregation patterns for the five model organisms had been determined to identify specific cell-cell interactions that might play a role during biofilm formation (Table 1). When mixed in 1/10 diluted Todd Hewitt Broth (THB; pH 7), which was also used as the flow medium, cells of *A. naeslundii* AK6 and *S. mitis* SK24 formed clearly visible, slowly settling coaggregates within 15 min (grade 2 according to the classification of Cisar [44]). These coaggregates had increased in size after 2 h and settled more rapidly, leaving the supernatant fluid only slightly turbid (grade 3). When *A. naeslundii* AK6 was mixed with *S. sanguinis* SK150, small uniform aggregates that remained in suspension formed within 2 h (grade 1). In contrast, *S. oralis* SK248 and *S. downei* HG594 did not form coaggregates with any of the other strains. In the biofilms, both organisms represented only minor fractions of the total biovolume. Cells of *S. oralis* SK248, which was inoculated first, were typically found scattered throughout the biofilm (Figure 1D), while cells of *S. downei* HG594, inoculated fourth, usually appeared in groups of particularly long chains intertwined with other streptococci (Figure 1E).

**Table 1:** Pairwise bacterial coaggregation.

	*S. oralis*			*S. sanguinis*			*S. mitis*			*S. downei*		
	t1	t2	t3	t1	t2	t3	t1	t2	t3	t1	t2	t3
***A. naeslundii***	0	0	0	0	1	1	2	3	3	0	0	0
***S. downei***	0	0	0	0	0	0	0	0	0	-	-	-
***S. mitis***	0	0	0	0	0	0	-	-	-	-	-	-
***S. sanguinis***	0	0	0	-	-	-	-	-	-	-	-	-

Pairwise coaggregation of *A. naeslundii* AK6, *S. downei* HG 594, *S. mitis* SK24, *S. sanguinis* SK150 and *S. oralis* SK248. Coaggregation was determined 15 min, 2 h and 24 h after mixing in 1/10 diluted Todd Hewitt Broth. t1 = 15 min; t2 = 2 h; t3 = 24 h. Grade 0: No visible aggregates in cell suspension. Grade 1: Small uniform aggregates in suspension. Grade 2: Definite coaggregates easily seen, but suspension remained turbid without immediate settling of coaggregates. Grade 3: Large coaggregates which settled rapidly leaving some turbidity in the supernatant fluid. Grade 4: Clear supernatant fluid and large coaggregates which settled immediately.

The bacterial composition of the biofilms proved to be highly reproducible in two biological replicates. For composition analysis, all examined biofilms were hybridized simultaneously with probe EUB338, targeting *Bacteria*, and a specific probe targeting one of the five employed organisms. Digital image analysis was then used to estimate the absolute biovolume visualized with each probe in each biofilm, and the relative biovolume visualized with the specific probe was determined. For *S. mitis* SK24, *S. sanguinis* SK150 and *A. naeslundii* AK6, both the absolute bacterial volumes and the relative biovolume fraction did not differ significantly between replicates. For *S. oralis* SK248 and *S. downei* HG594, absolute bacterial volumes were lower in the second replicate than in the first (P<0.05), but so were the absolute volumes determined with EUB338. The relative biovolume fractions were not statistically different between the first and second replicate for both *S. oralis* SK248 and *S. downei* HG594. Detailed biofilm composition data are shown in Table 2 and Figure S1.

**Table 2:** Bacterial composition of the model biofilms.

Organism	BF Replicate 1	BF Replicate 2	Total BF
*S. oralis* SK248	0.35%	0.17%	0.29%
*A. naeslundii* AK6	2.89%	2.62%	2.76%
*S. mitis* SK24	76.3%	79.2%	77.6%
*S. downei* HG594	0.93%	0.62%	0.81%
*S. sanguinis* SK150	14.2%	10.6%	10.9%

Biovolume fractions of *S. oralis* SK248, *A. naeslundii*, *S. mitis* SK24, *S. downei* HG594 and *S. sanguinis* SK150, as determined with FISH and digital image analysis. BF: Biovolume Fraction.

### pH landscapes in the model biofilm

**Figure 2:**
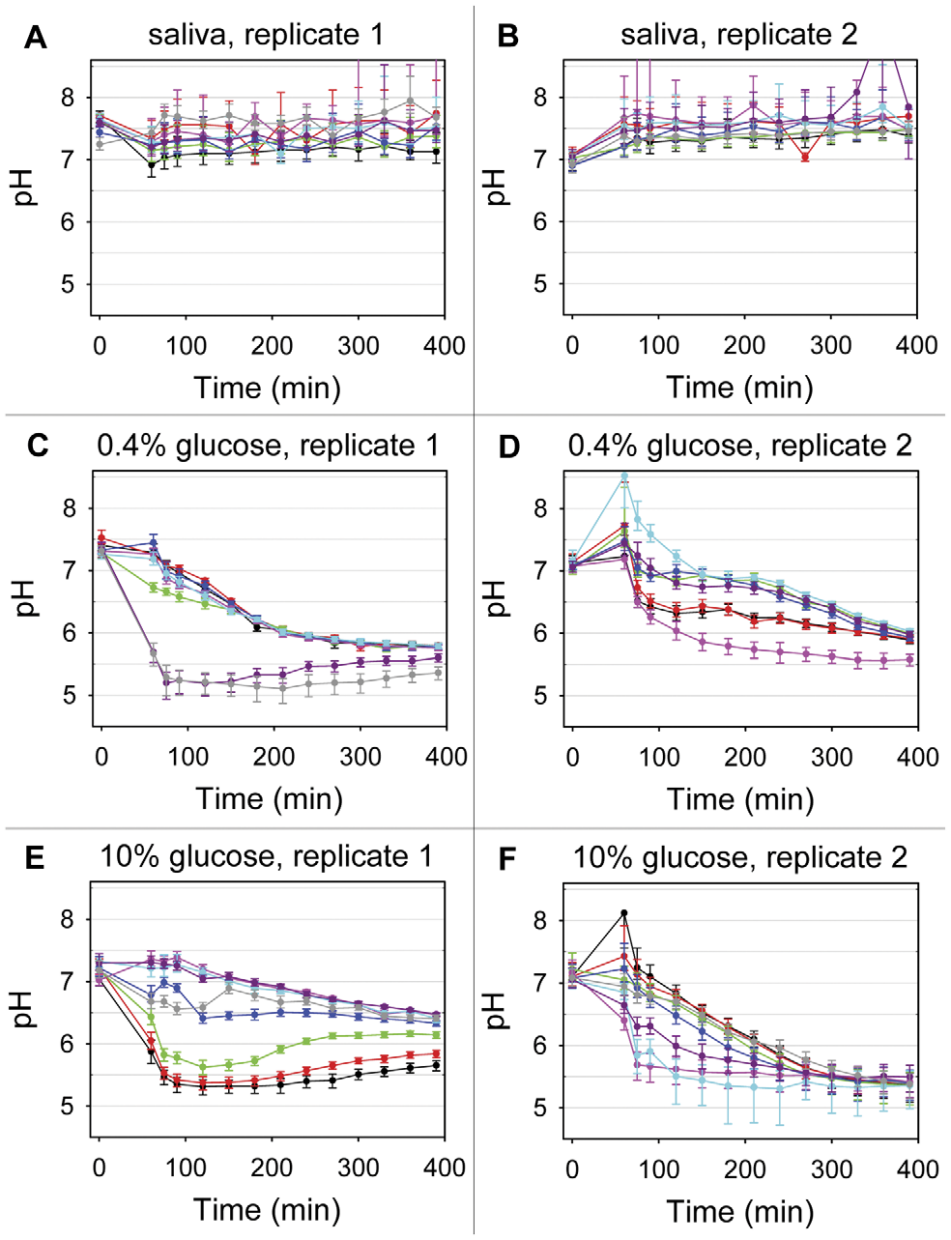
Extracellular pH in the model biofilms. Extracellular pH in the bottom layer of the biofilms was determined using the ratiometric probe C-SNARF-4 and confocal microscopy. In each biofilm, 8 different fields of view were monitored for 390 min. Each line graph represents the average pH in one field of view (143 µm^2^). **A** and **B**: When exposed to saliva without glucose, extracellular pH in the biofilms remained continuously above 7. **C**, **D**, **E** and **F**: Upon addition of saliva containing 0.4% (**C** and **D**) or 10% glucose (**E** and **F**) (t = 60 min), pH drops occurred in all examined microscopic fields. Decreases in pH varied considerably in different areas of the biofilms, and distinct pH microenvironments persisted for several hours. Error bars indicate standard deviations.

**Figure 3:**
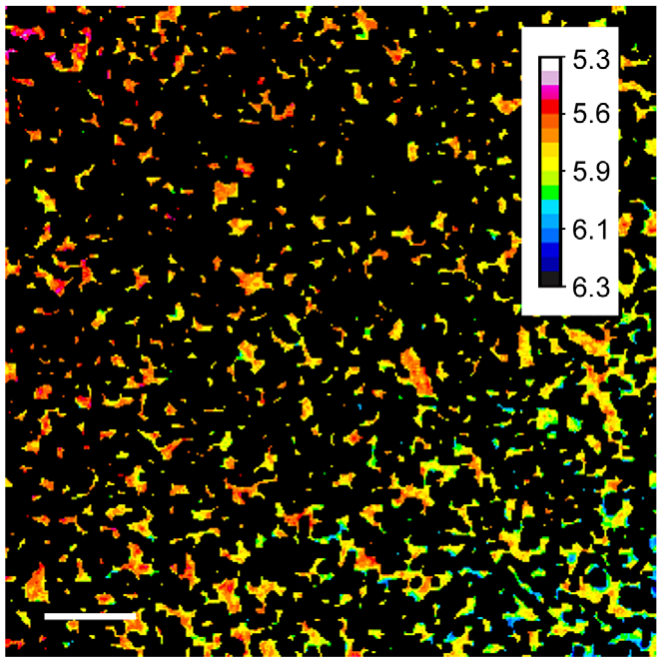
Extracellular pH gradients in the model biofilms. A biofilm was exposed to sterile saliva containing 0.4% (w/v) glucose and the ratiometric pH-sensitive dye C-SNARF-4. Bacterial cells were removed from the image, and extracellular pH was determined. False colours have been used to represent the local pH as shown in the scale bar appended to the image. The higher cell density in the top left part of the picture is correlated with lower pH values, as compared to the bottom right part. Bar = 20 µm.

Biofilms were incubated with sterile saliva containing 0%, 0.4% or 10% glucose, and pH in the bottom layer of each biofilm was monitored in eight different microscopic fields of view for 330 min. In the presence of pure saliva, no acid production occurred, and the pH remained constantly above 7 until the end of the observation period (Figure 2A, B). Upon addition of sterile saliva containing 0.4% glucose, acid production in the biofilms started immediately. Interestingly, the speed with which pH decreased varied considerably between different fields of view. In some areas of the biofilms, pH fell quickly to values below 5.5, while in others, acid production was slower and yielded a final pH of 5.7–5.9 (Figure 2C, D). Approximately one hour after the addition of glucose, a certain pH equilibration between different fields of view could be observed. Still, pH values in different areas of the biofilms remained distinct over the entire period of 330 min. In phases of high acid production, image analysis revealed pH gradients even within single fields of view (Figure 3).

Biofilms exposed to sterile saliva containing 10% glucose showed a similar behaviour. In some areas, rapid pH drops to 5.5 and slightly below occurred, while in others, acid production was slow with final pH values of up to 6.5 (Figure 2E, F). Again, distinct pH-environments were conserved over several hours, although all fields of view were in close proximity (0.5 mm–5 mm). The biofilm thickness in different fields of view varied between 7 µm and 100 µm, but we did not observe an obvious correlation between thickness and pH drop in the bottom layer (data not shown).

**Figure 4:**
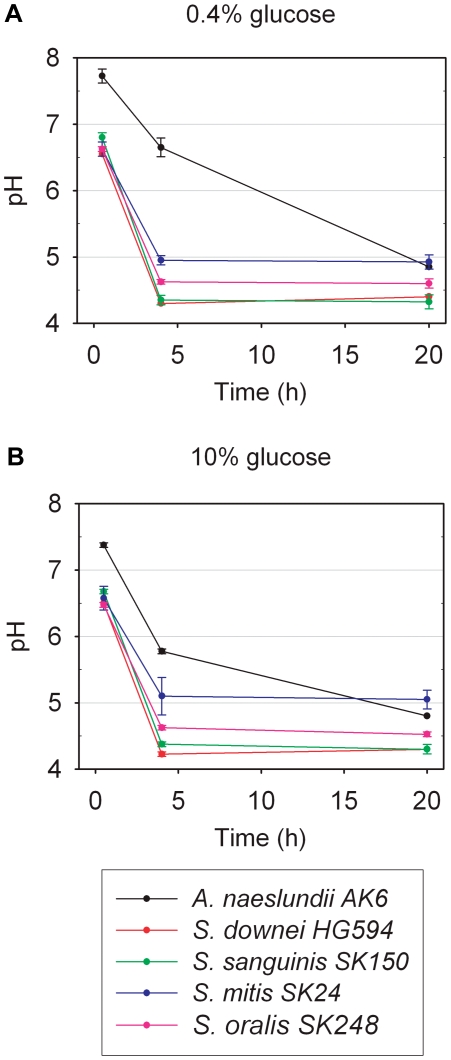
pH in planktonic suspension. **A.** Overnight cultures of *S. oralis* SK248, *A. naeslundii* AK6, *S. mitis* SK24, *S. downei* HG594, and *S. sanguinis* SK150 were washed twice and suspended in sterile saliva containing 0.4% (w/v) glucose. pH was measured after 30 min, 4 h and 20 h. All *Streptococcus* spp. reached terminal pH within four hours. pH drops in suspensions of *A. naeslundii* occurred more slowly. **B.** When glucose concentration was raised to 10% (w/v), no differences in terminal pH were observed. After 20 h, pH was lowest for *S. sanguinis* and *S. downei* (4.3–4.4) and highest for *S. mitis* SK24 (4.9–5). In suspensions of *A. naeslundii*, pH dropped more quickly as compared to 0.4% glucose. Experiments were performed in duplicate and repeated on another day. Error bars indicate standard deviations.

Before the five organisms were used in biofilm experiments, their acid production in planktonic culture was tested. Terminal pH values ranged between 4.3 for *S. sanguinis* SK150 and 5.0 for *S. mitis* SK24 after 20 h of incubation in saliva containing 0.4% or 10% glucose (Figure 4). When the pH value in a field of view approached the terminal pH of *S. mitis* SK24, the predominant organism of the biofilm, acid production diminished and the pH increased slightly, probably due to the buffering capacity of the overlying fluid (Figure 2C, E). Staining with the BacLight bacterial viability kit indicated that the majority of all organisms remained viable in the course of an experiment (Figure S2).

## Discussion

We developed and validated a laboratory dental biofilm model that exclusively employed mildly acidogenic human oral bacteria. It is, to our knowledge, the first to specifically mimic the bacterial community that is present during early acidogenic stages of the caries process. The bacterial composition in the model proved to be reproducible and bore resemblance to the microflora observed *in vivo*. When stimulated with glucose, the model biofilms created a mildly acidic environment, similar to the one expected in young dental biofilms. Importantly, different pH-microenvironments formed in the extracellular matrix at the base of the biofilms and were conserved over several hours.

Sequential inoculation of the organisms into the flow channels permitted the best possible standardization of biofilm growth. *S. oralis* SK248 and *A. naeslundii* AK6 were injected first and second, because they belong to the earliest colonizers of the human tooth [26], [27]. Moreover, recent investigations have reported a symbiotic relationship between strains of both species in a flow cell model [9], [45]. As *S. mitis* SK24 coaggregates with *A. naeslundii* AK6 (Table 1), the organism was inoculated third. *S. downei* HG594 and *S. sanguinis* SK150 were the most acidogenic bacteria in planktonic culture (Figure 4), and were therefore injected last in order to avoid overgrowth by these organisms.

The flow rate during growth (0.47 mm/s) was set within the range of flow speeds estimated for salivary film in the human oral cavity (0.013–5.833 mm/s) [46]. While a medium based on human saliva best reflects the environment in the oral cavity, it is difficult to obtain and to standardize. We therefore used 1/10 diluted THB (pH 7; 0.02% glucose) as the flow medium during biofilm growth. However, THB was replaced with sterile saliva for the subsequent analysis of extracellular pH. Biofilms were not grown on human enamel, as the use of plastic flow cells provided the advantage of direct microscopic examination of basal layers of the biofilms through the bottom of the flow cell. The closed system of only five bacterial species permitted to design specific FISH probes for all organisms and to determine the bacterial composition of the biofilms using digital image analysis. Although this approach did not provide information about the viability of single organisms, it offered the possibility to describe the biofilm architecture and to study colony patterns of the employed strains directly *in situ*.

The topography of our model biofilms matched that of *in-vivo*-grown dental biofilms (Figure 1, Video S1), and the thickness (7–100 µm) was similar to that of one-day-old human smooth surface plaque [47]. In two replicates, the bacterial composition of the biofilms was very similar, with *S. mitis* SK24 and *S. sanguinis* SK150 constituting most of the biovolume (Table 2, Figure S1). Both species have also been reported to be dominant in early dental biofilms [25], [48]. In contrast, the MS *S. downei* HG594 represented only a small fraction of the biofilm, which is also typical for early dental plaque [25], [48] and might be explained by the constant pH of 7 in the growth medium [4]. *S. oralis* is a frequent isolate from both young and mature plaque [26], [27], [49], but it was outcompeted in our model and accounted for less than 0.3% of the biovolume. While all streptococcal species were distributed uniformly across the different layers of the biofilms, *A. naeslundii* AK6 was predominantly detected at the biofilm base, where it formed branched, sometimes spider-like colonies (Figure 1E, Video S1). It is conceivable that the coaggregation observed in planktonic culture between *A. naeslundii* AK6 and *S. mitis* SK24 played a role during biofilm formation, in that attached cells of *A. naeslundii* facilitated the adhesion of *S. mitis*. Interestingly, the growth pattern of *A. naeslundii* in our model biofilm shows striking similarity to the one determined in a recent investigation of initial human plaque grown *in vivo* (Figure S3) [28].

To our knowledge, this is the first study that provides a detailed time-resolved microscopic analysis of the extracellular pH landscape at the interface between a dental plaque model biofilm and the underlying substrate. While previous studies employing microelectrodes have demonstrated the presence of pH gradients between surface and bottom of mature dental biofilms (200 µm–6 mm) [40], [41], we were able to visualize horizontal pH gradients in the basal layer of young dental caries model biofilms (7–100 µm). Upon addition of glucose, the salivary medium became anoxic (Figure S4), and the pH decreased at different rates in different areas of the biofilms. The resulting pH microenvironments, although in close spatial proximity, persisted for several hours (Figure 2). These differences might reflect variations in bacterial composition or indicate that the bacteria were in different states of metabolic activity in different areas of the biofilms. Aerobic degradation of lactate, which has been reported for various strains of *A. naeslundii* [50], might also initially have contributed to the establishment of different micro-niches. When pH reached values close to the terminal pH of *S. mitis* SK24, the dominant organism in the biofilms, the pH decrease stopped. The acidogenic metabolic processes were probably inhibited at this pH, although the cell viability seemed unaffected (Figure S2).

pH-sensitive ratiometric fluorescent dyes are a promising tool for the microscopic analysis of pH microenvironments in bacterial biofilms. Unlike pH-microelectrodes, they do not mechanically perturb the biofilms, and they provide an unsurpassed spatial resolution in both the XY and Z planes. The ratio between the fluorescent emissions of the protonated and the unprotonated dye is uniquely dependent on the local pH, and the analysis is therefore not affected by probe compartmentalization or differences in probe penetration, which are likely to occur in biofilms. Moreover, exact quantitative analysis is not hampered by photobleaching and differences in laser power or optical path length.

Some studies on eukaryotic cells have reported that the calibration of ratiometric dyes is affected by charge-charge interactions with biological compounds [51], [52]. It is therefore a potential problem that calibration cannot be performed directly in the sample matrix, but is done separately in titrated buffer solutions. However, Hunter *et al.* [42], who were the first to investigate pH environments in bacterial biofilms using C-SNARF-4, incubated the dye with different concentrations of the highly charged bacterial exopolysaccharide alginate, with bovine serum albumin and with cells of *P. aeruginosa*, and concluded that none of these biomaterials influenced the fluorescent emission characteristics of C-SNARF-4 at pH<7. These results suggest that matrix components of the model biofilms did not affect the results obtained with C-SNARF-4 in our study.

It is crucial to only consider the pH in the extracellular matrix when analysing dental biofilms, as the intracellular bacterial pH will not affect de- or remineralisation of the tooth. Control staining of our model biofilms with SYTO9 showed that C-SNARF-4 was taken up and even concentrated by all cells (Figure S5). This permitted to employ C-SNARF-4 as the only dye and to use digital image analysis to remove all organisms from the ratiometric pictures before pH was determined (Figure S6). Moreover, by using only one dye for both extracellular pH calculation and bacterial biomass detection, any influence of additional stains on fluorescent emission in the C-SNARF-4 detection windows could be excluded.

The presented biofilm model is the first to mimic the bacterial community present in early acidogenic stages of dental caries. Its high reproducibility and structural similarity to *in-vivo*-grown biofilms make it a suitable tool to investigate the effect of potential therapeutic agents on biofilm development and composition, as well as on the extracellular pH at the biofilm-substratum interface. Staining with the ratiometric probe C-SNARF-4 and direct confocal microscopic examination of the flow cells allow for a thorough real-time analysis of the heterogeneous pH landscape in the biofilms in all three dimensions. This offers valuable insight into the pathogenic process leading to tooth decay, and might, beyond that, contribute to our general understanding of metabolic processes in bacterial biofilms.

## Materials and Methods

### Bacterial strains

*Streptococcus oralis* SK248, *Streptococcus mitis* SK24, *Streptococcus sanguinis* SK150 and *Actinomyces naeslundii* AK6 were kindly provided by M. Kilian, Institute of Medical Microbiology and Immunology, Aarhus University, Denmark. *Streptococcus downei* HG594 was kindly provided as *Streptococcus mutans* HG594 by J. J. de Soet, Academic Centre for Dentistry, Amsterdam, Netherlands, and reclassified as *S. downei* after 16S sequence analysis. All strains were human oral isolates. Organisms were cultivated aerobically on blood agar (SSI, Copenhagen, Denmark) and transferred to THB (Roth, Karlsruhe, Germany) at 35°C until mid to late exponential phase prior to experimental use (Figure S7).

### 16S rRNA gene sequencing

The 16S rRNA gene of the selected bacterial strains was amplified using two different primer sets, 8F/1492R and EUB26F/1390R, both specific for the domain *Bacteria* (see Table S1). Reaction mixes contained 50 µl of Taq DNA Polymerase Master Mix RED (Ampliqon, Copenhagen, Denmark), 46 µl of distilled water, 2 µl each of forward and reverse primers (10 pmol/µl; MWG Biotech, Ebersberg, Germany), and template DNA from a single bacterial colony. Amplifications were carried out on a PTC-200 Peltier Thermal Cycler (MJ Research, Waltham, Massachusetts, USA) for 25 cycles (initial denaturation: 93°C, 5 min; denaturation: 92°C, 30 s; annealing: 52°C, 60 s; elongation: 72°C, 90 s; last elongation: 72°C, 10 min). Successful PCR amplification was confirmed by Agarose gel electrophoresis, and PCR products were purified using the GenElute™ PCR Clean-Up Kit according to the manufacturer's instructions (Sigma-Aldrich, Brøndby, Denmark). Sequencing was performed by Macrogen (Seoul, Korea) using the primers 8F, EUB 26F, EUB 518F, 907F, 907R, 1390R and 1492R (For primer sequences and references, see Table S1). All steps were performed in triplicate. Consensus sequences were submitted to GenBank (accession numbers: HQ219654–HQ219658).

### Coaggregation assays

Bacterial cells were harvested, washed, resuspended in 1/10 diluted THB (0.02% (w/v) glucose; pH 7.0) and adjusted to an optical density of 1.0 (550 nm). Aliquots of 0.2 ml were mixed and pair coaggregation was evaluated after 15 min, 2 h and 24 h according to the classification of Cisar [44]. Experiments were performed in triplicate and repeated twice.

### Biofilm growth

Flow cells (ibiTreat, μ-slide VI, Ibidi, Munich, Germany) were preconditioned with 1/10 THB (pH 7.0). Bacterial cultures, adjusted to an optical density of 0.4 at 550 nm (corresponding to 2–5*10^9^ cells/ml), were inoculated sequentially into the flow channels in the following order: 1. *S. oralis* SK248; 2. *A. naeslundii* AK6; 3. *S. mitis* SK24; 4. *S. downei* HG594; 5. *S. sanguinis* SK150. 0.4 ml of each organism was injected through the silicone tubing at the in-port using sterile needles (BD Microlance, 27G, Drogheda, Ireland), and injection holes were sealed with silicone glue (Dow Corning, Wiesbaden, Germany). Following injection, the flow was halted for 30 min to allow for bacterial adhesion. Nonadherent organisms were removed by 10 min of flow prior to injection of the next organism. After inoculation procedure, biofilms were grown for 26 h at 35°C under constant flow (250 µl/min; 28.3 mm/min) of 1/10 THB (pH 7.0) provided by a peristaltic pump (Watson Marlow 205 U, Wilmington, Massachusetts, USA).

### FISH probe design

The species-specific oligonucleotide probes SORA2, ANAES, SMIT, SDOW and SSAN, targeting the five organisms of the model biofilm, were designed. Probes were synthesized commercially (biomers.net, Ulm, Germany) and 5′ end-labelled with Cy3 (indocarbocyanine). Additionally, unlabelled helper probes SORA2H, ANAESH1, ANAESH2, SMITH1, SMITH2, SDOWH and SSANH were employed in FISH experiments (see Table S2 for probe sequences). The probes were checked for their practical use in hybridization experiments using the program OLIGO (version 7.0; Cascade, Colorado, USA). The stringencies of all probes were adjusted according to a previously described protocol [53]. Briefly, fixed cells of each strain and a negative control organism were subjected to FISH using hybridization mixes with formamide concentrations ranging from 0% (v/v) to 75% (v/v). Pictures with fixed exposure times were acquired with a wide field microscope (Zeiss Axiovert 200 M, Jena, Germany) equipped with a 100 W high-pressure mercury lamp (HB103, Osram, Winterthur, Switzerland). The software daime [54] was used to compare the signal intensities emitted by target and non-target cells at different concentrations of formamide (Figure S8). All subsequent FISH experiments were carried out with hybridization buffers containing 10% (v/v) of formamide. Probe EUB338 [55] was 5′ labelled with Atto633 (biomers.net, Ulm, Germany) and used to visualize all bacteria in the biofilms. Probe NONEUB [56], labelled with Atto633, was employed in control experiments to check for unspecific probe binding.

### FISH

After biofilm growth, the flow medium was removed by aspiration with thin paper points. Biofilms were fixed in the flow cells for 1 h at 4°C in 4% paraformaldehyde, washed twice with PBS, dried in an ethanol series (50%, 80% and 96% for 3 min), and stored at 4°C. Before hybridization, bacterial cells were permeabilized with lyzozyme (1 mg/ml) at 37°C for 10 min. *In situ* hybridizations were carried out in isotonically equilibrated humidity chambers at 46°C for 3 h. 100 µl of hybridization buffer (0.9 M NaCl, 20 mM Tris/HCl pH 7.5, 0.01% SDS, 10% formamide; 100 ng of the designated oligonucleotide probes) was applied per channel. Subsequently, two stringency washes were performed at 50°C for 15 min using washing buffer containing 20 mM Tris/HCl pH 7.5, 5 mM EDTA, 0.01% SDS and 112 mM NaCl. The buffer was removed by aspiration, and 60 µl Citifluor/Vectashield Hard Set (4/1; Citifluor Ltd., London, UK; Vectashield, Burlingame, California, USA) was added.

In all FISH experiments, fixed cells of *S. downei* HG594 were included as negative controls for probes ANAES (11 mismatches), SMIT (8 mismatches) and SSAN (8 mismatches), while *S. mitis* SK24 and *S. sanguinis* SK150 served as negative controls for probes SORA2 (5 mismatches) and SDOW (7 mismatches), respectively. At a formamide concentration of 10% (v/v), all specific probes visualized the target organisms, but not the negative controls. Probe EUB338 detected all cells in the biofilms (Figure S9). Control hybridization of biofilms with probe NONEUB confirmed that the fluorescent signals did not derive from unspecific binding or autofluorescence (data not shown).

### Biofilm composition analysis

Six biofilms were grown simultaneously. Five of them were subjected to FISH with EUB338 and either SORA, ANAES, SMIT, SDOW or SSAN. The sixth biofilm was subjected to control hybridization using NONEUB. An inverted confocal microscope (Zeiss LSM 510 META, Jena, Germany) equipped with a 63× oil immersion objective, 1.4 numerical aperture (Plan-Apochromat) was used for biofilm composition analysis. 543 nm and 633 nm laser lines were used for Cy3 and Atto633 excitation. Fluorescence emission was detected in 565–615 nm and 651–704 nm intervals (META detector) with the confocal pin hole set to 1 Airy unit (0.8 µm optical slice thickness). For each flow channel, 16 fields of view were chosen at random and Z-stacks consisting of six equispaced XY focal planes spanning the height of the biofilms were acquired. Images were 1320×1320 pixels (143×143 µm^2^) in size and were acquired with pixel dwell time 2.5 µs, line average 2, 0.11 µm/pixel, 12-bit intensity resolution. Two replicate series of six biofilms were grown and examined independently. The program daime [54] was employed to determine the area of the bacterial mass visualized by EUB338 and the respective species specific probe in each microscopic image. For each stack of confocal images, the biovolume of the entire bacterial population was estimated by multiplying the total area visualized by EUB338 with the distance between the layers of the stack according to the Cavalieri principle [57]. Likewise, the biovolumes for each of the five bacterial species detected by specific probes were estimated.

### Statistical analysis of the biofilm composition data

Unpaired Student's t-tests were carried out to assess differences of the absolute and relative biovolumes between the first and the second biological replicate. P-values below 0.05 were considered statistically significant.

### Acid production assays with planktonic bacteria

Pooled stimulated saliva was filter sterilized using the method described by de Jong *et al.* [58]. The saliva was kept on ice before filtration and then stored at −20°C until used in the experiments. Bacteria were harvested, washed twice and resuspended in sterile saliva. Optical density was adjusted to 1.0 at 550 nm, glucose was added to concentrations of 0.4% (w/v) or 10% (w/v), and bacterial suspensions were incubated at 35°C for 20 h. pH was measured after 30 min, 4 h and 20 h. Experiments were performed in duplicate for all organisms and repeated on another day.

### Oxygen concentration measurements

Biofilms were grown as described above. An oxygen microelectrode, fabricated as described by Revsbech [59], with a tip diameter of 30 µm, was fixed inside a syringe tip (Becton Dickinson S.A., Madrid, Spain) with UV-curing cement (Loctite 190672, Henkel, Taastrup, Denmark). After biofilm growth phase, the microelectrode was positioned in the outport of the flow channel in close proximity to the biofilm, and flow of sterile saliva containing 10% (w/v) glucose was applied until the growth medium was replaced. During saliva flow, the measured oxygen concentration was close to air saturation. When flow was stopped, the oxygen concentration decreased and the saliva medium in the flow channels became completely anoxic within one hour (Figure S4). The program Sensor Trace Basic V.2.1 (Unisense, Aarhus, Denmark) was used to record electrode data. Experiments were performed in duplicate.

### Ratiometric pH measurement

For confocal microscopic calibration, HEPES buffer solutions (50 mM; adjusted to pH 4.5–8.5 in steps of 0.1 pH units), containing C-SNARF-4 at a concentration of 30 µM, were imaged in flow channels. A Zeiss LSM 510 META (Jena, Germany) with a 63× oil immersion objective, 1.4 numerical aperture (Plan-Apochromat) was used for image acquisition. The probe was exited with a 543 nm laser line (250–300 µW), and fluorescence emission was monitored simultaneously within 576–608 nm (green) and 629–661 nm (red) intervals (META detector), with the pin hole set to 2 Airy units (1.6 µm optical slice thickness). Images were 364×364 pixels (143×143 µm^2^) in size and were acquired with pixel dwell time 18 µs, line average 2, 0.4 µm/pixel (zoom 1), 12-bit intensity resolution. For every third pH value, a measurement was performed on unstained HEPES buffer (50 mM, pH 8.5) for background subtraction. Additionally, unstained solutions of glucose (20% w/v) and lactate (20% w/v), 1/10 diluted THB (pH 7), sterile saliva, PBS (pH 7.4, Sigma-Aldrich, Brøndby, Denmark) and untreated biofilms were imaged with identical microscope and laser settings. No autofluorescent signals were emitted by any of these controls in the wavelength ranges 576–608 nm and 629–661 nm.

For ratio calculation, regions of 100×100 pixels were defined within each image and the average and standard deviations were determined using the LSM acquisition software. Subsequently, the ratio *R*, standard deviation *s_R_* and standard error of mean, *S_R_*, were calculated for each pH value according to equations (1), (2) and (3):
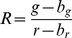
(1)

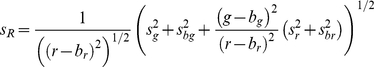
(2)

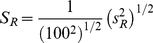
(3)
*g*, *r*, *s_g_* and *s_r_* are the averages and standard deviations within the 100×100 pixels region defined in the respective green and red images. *b_g_*, *b_r_*, *s_bg_* and *s_br_* are the corresponding values for the background images. The resulting values of *R* were plotted in MATLAB (MathWorks, Natick, Massachusetts, US), and fitted to the function:
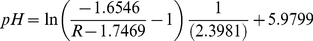
(4)Measurements were performed twice and proved to be highly reproducible. The calibration data and fitted curve are shown in Figure S10.

For ratiometric pH-analysis, three biofilms were grown in parallel. After 30 h, growth medium was removed applying 45 min of PBS flow, and the flow cells were transferred to the microscope, which was kept at 37°C with an XL incubator (PeCon, Erbach, Germany). Eight fields of view were chosen at random in each channel, and XY positions were marked in the LSM software. C-SNARF-4 was added to the PBS (30 µM final concentration) and baseline pH images were acquired in the bottommost layer of the biofilms (t = 0 min). PBS was then removed by carefully sucking the liquid from each flow channel by insertion of paper points at the end of the channel. The flow channels were washed twice with sterile saliva, and finally filled with 100 µL salivary solutions containing 30 µM C-SNARF-4 and 0% (w/v), 0.4% (w/v) or 10% (w/v) glucose (t = 60 min). Identical fields of view were followed over a total time period of 390 min. pH images were acquired at t = 60 min, t = 75 min, t = 90 min and then in intervals of 30 min. For background subtraction, images were acquired with the 543 nm laser switched off. The microscope and laser settings were identical to those of the calibration measurements. All experiments were performed in duplicate.

### Viability staining

To assess viability of the bacteria in the course of pH experiments, additional biofilms were stained with BacLight according to the manufacturer's instructions (Invitrogen, Taastrup, Denmark). Viability was assessed immediately after growth phase and at the end of an experiment. A Zeiss LSM 510 META (Jena, Germany) with a 63× oil immersion objective, 1.4 numerical aperture (Plan-Apochromat) was used for image acquisition. Excitation was performed with 488 nm and 543 nm laser lines. Emission was detected with the META detector set to 500–554 nm and 554–608 nm, respectively, and the pin hole set to 2 Airy units (1.6 µm optical slice thickness). Fields of view were chosen at random, and Z-stacks spanning the height of the biofilms were acquired. Images were 364×364 pixels (143×143 µm^2^) in size and were acquired with pixel dwell time 18 µs, line average 2, 0.4 µm/pixel, 12-bit intensity resolution.

### pH data analysis

C-SNARF-4 was taken up and concentrated by the bacteria employed in the model. To check if all organisms could be visualized with the help of C-SNARF-4 alone, some flow channels were dried carefully following pH data acquisition and biofilms were stained with SYTO9 (Invitrogen, Taastrup, Denmark). As all organisms detected by SYTO9 were equally visualized with C-SNARF-4 (Figure S5), no additional stain was used for subsequent image analysis.

In order to exclusively determine the extracellular pH in the biofilm, the program daime was employed to remove the entire bacterial biomass from all pictures (Figure S6). In ImageJ (http://rsb.info.nih.gov/ij), background fluorescence was subtracted, the “mean” filter (radius: 1 pixel) was employed to compensate for detector noise, and the ratio between fluorescence intensities detected with the green (576–608 nm) and red (629–661 nm) filter sets was determined for every extracellular pixel. Average ratios and standard deviations were calculated for each field of view and converted to pH values according to equation (4).

## Supporting Information

Figure S1 **Detailed biovolume fractions for each organism in the model biofilms.** Each circle represents one microscopic field of view. Bars indicate means. **A.** *S. oralis* SK248. **B.** *A. naeslundii* AK6. **C.** *S. mitis* SK24. **D.** *S. downei* HG594. **E.** *S. sanguinis* SK150.
(TIF)

Figure S2 **Viability of the organisms in the biofilm before and after pH-response experiments.** Biofilms were stained with the BacLight viability kit before (**A**) and after (**B**) pH-response experiments. Viable bacteria appear green and membrane-compromised bacteria red. No changes in viability could be observed in the course of a 6.5 h experiment. Bars = 20 µm.
(TIF)

Figure S3 **CLSM images of** ***in situ*** **multilayered dental biofilm.** The biofilms were hybridized simultaneously with all-bacterium-specific probe EUB338, *Streptococcus*-specific probe STR405 and *Actinomyces naeslundii*-specific probe ACT476. Yellow-green, blue and red represent streptococci, *A. naeslundii* and other bacteria, respectively. **A.** XY section in the basal layer of a 24-h biofilm showing spider-like colonies of *A. naeslundii* in a *streptococcus*-dominated biofilm. Bar = 25 µm. **B.** XZ section of 48-h biofilm. Note the preferential location of *A. naeslundii* in the inner part of the biofilm next to the surface (bottom of the image). Image width = 200 µm. Images from Dige I. Initial dental biofilm formation studied by confocal laser scanning microscopy and fluorescence *in situ* hybridization. Faculty of Health Sciences, Aarhus University. PhD dissertation (2008).
(TIF)

Figure S4 **Oxygen concentration in the outport of the flow channel.** During flow of sterile saliva containing 10% (w/v) glucose, oxygen concentration in the effluent saliva was close to air saturation. When flow was stopped (t = 4 min), the oxygen concentration decreased until anoxic conditions were reached.
(TIF)

Figure S5 **Staining of biofilms with C-SNARF-4 and SYTO9.** Both pictures show identical fields of view. Biofilm was first stained with C-SNARF-4 (**A**), then with SYTO9 (**B**). All cells detected with SYTO9 were equally visualized with C-SNARF-4. Bars = 20 µm.
(TIF)

Figure S6 **Removal of cells stained with C-SNARF-4 from biofilm images A.** Biofilm stained with C-SNARF-4. **B.** The program daime was employed to remove all cells from the pH data pictures in order to exclusively determine extracellular pH in the biofilm. Bars = 20 µm.
(TIF)

Figure S7 **Growth of the bacterial strains in planktonic culture.** **A.** *S. oralis* SK248. **B.** *A. naeslundii* AK6. **C.** *S. mitis* SK24. **D.** *S. downei* HG594. **E.** *S. sanguinis* SK150. Bacteria were grown aerobically in Todd Hewitt Broth at 35°C. Experiments were performed in triplicate. Error bars indicate standard deviations.
(TIF)

Figure S8 **Optimization of FISH conditions for the designed oligonucleotide probes.** Probes were hybridized to fixed cells of the positive and negative controls, using varying formamide concentrations in the hybridization buffer. Images with fixed exposure time were acquired, and the signal intensities were measured and given as RU = relative fluorescent units. **A.** Probe SORA2+helper probe SORA2H. **B.** Probe ANAES+helper probes ANAESH1 and ANAESH2. **C.** Probe SMIT+helper probes SMITH1 and SMITH2. **D.** Probe SDOW+helper probe SDOWH. **E.** Probe SSAN+helper probe SSANH. Even at low concentrations of formamide the signal from the negative controls was weak. All subsequent FISH experiments were performed at 10% formamide.
(TIF)

Figure S9 **Specificity of FISH probes.** Fixed cells of positive and negative control organisms were submitted to FISH with EUB338 (magenta) and the species-specific probes SORA2, ANAES, SMIT, SDOW or SSAN (red). Positive controls *S. oralis* SK248, *A. naeslundii* AK6, *S. mitis* SK24, *S. downei* HG594 and *S. sanguinis* SK150 were detected with EUB338 (**A**, **E**, **I**, **M**, **Q**) and with the respective specific probes SORA2 (**C**), ANAES (**G**), SMIT (**K**), SDOW (**O**) and SSAN (**S**). Negative controls were detected with EUB338 (**B**, **F**, **J**, **N**, **R**), but not with the respective specific probes (**D**, **H**, **L**, **P**, **T**). Bars = 10 µm.
(TIF)

Figure S10 **Calibration of C-SNARF-4.** Black diamonds show green/red-ratios of C-SNARF-4 fluorescent emissions at different pH values, as determined in HEPES buffer. A sigmoid function was fitted to the ratios and used as calibration curve for the biofilm experiments.
(TIF)

Video S1 **Biofilm hybridized with probes ANAES (green) and EUB338 (red).** EUB338 visualizes the entire bacterial population, while ANAES specifically detects *A. naeslundii*. Cells of *A. naeslundii* predominantly colonize basal layers of the biofilm and are rarely observed in the middle and upper third. Field of view size: 143×143 µm. Biofilm height: 29.5 µm.
(WMV)

Table S1 **Sequences of primers used for PCR amplification and sequencing.**
(DOC)

Table S2 **Sequences of newly designed oligonucleotide probes.**
(DOC)
