# Supplementary figures and images for: pH Landscapes in a Novel Five-Species Model of Early Dental Biofilm
Source: PLoS One. 2011 Sep 23;6(9):e25299. doi: 10.1371/journal.pone.0025299 (PMC3179500; doi:10.1371/journal.pone.0025299)

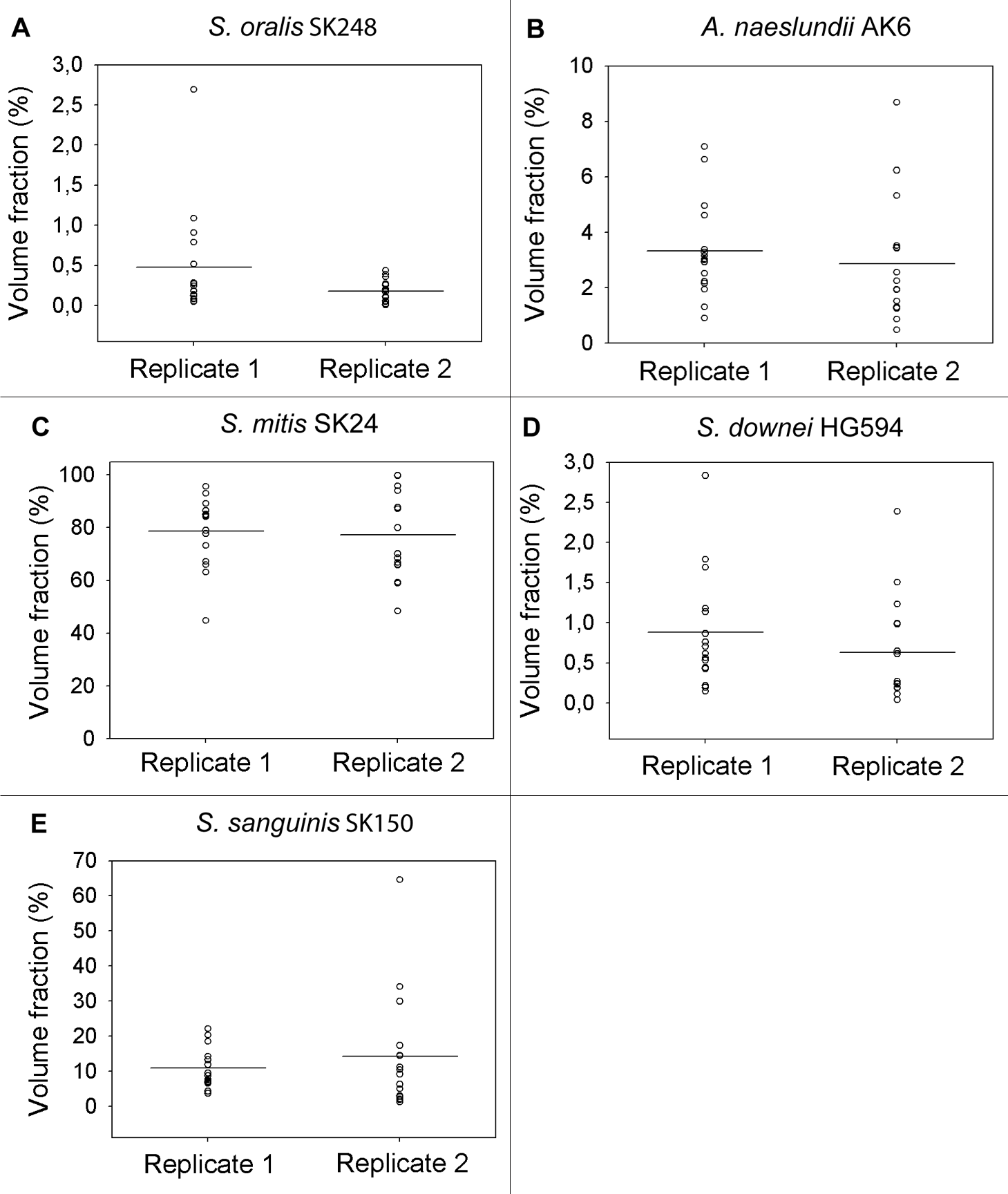

Supplement: Figure S1 — Detailed biovolume fractions for each organism in the model biofilms. Each circle represents one microscopic field of view. Bars indicate means. A. S. oralis SK248. B. A. naeslundii AK6. C. S. mitis SK24. D. S. downei HG594. E. S. sanguinis SK150. (TIF) [file pone.0025299.s001.tif]

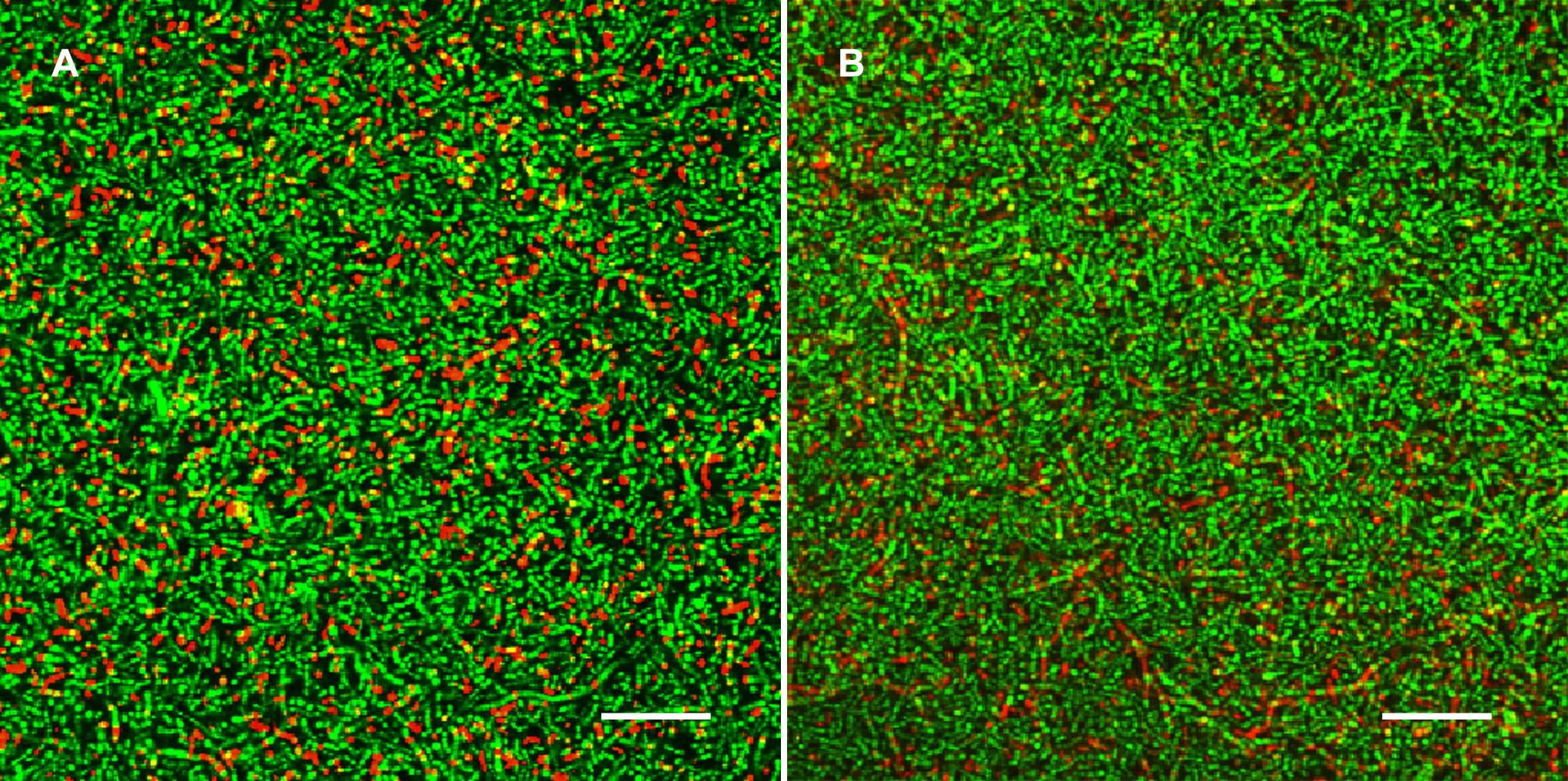

Supplement: Figure S2 — Viability of the organisms in the biofilm before and after pH-response experiments. Biofilms were stained with the BacLight viability kit before (A) and after (B) pH-response experiments. Viable bacteria appear green and membrane-compromised bacteria red. No changes in viability could be observed in the course of a 6.5 h experiment. Bars = 20 µm. (TIF) [file pone.0025299.s002.tif]

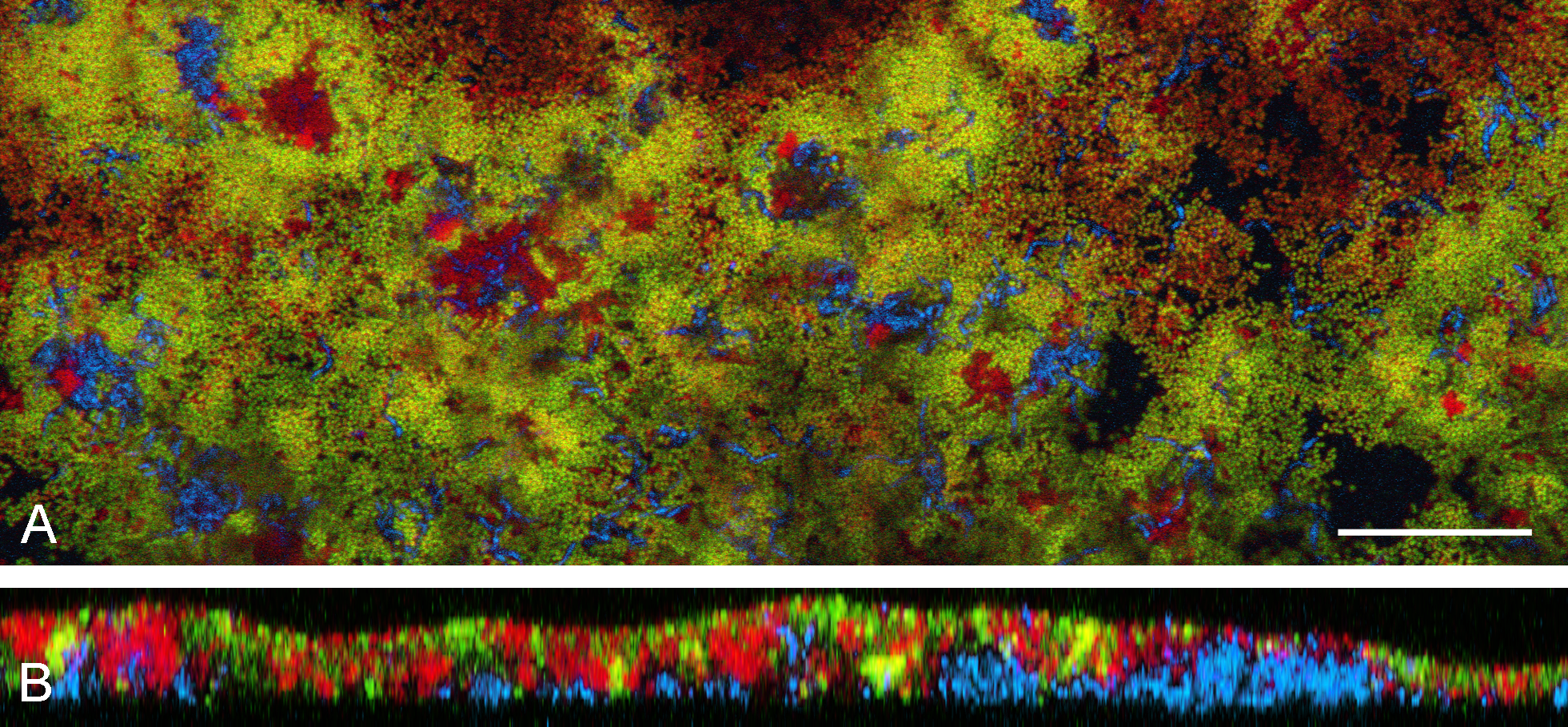

Supplement: Figure S3 — CLSM images of in situ multilayered dental biofilm. The biofilms were hybridized simultaneously with all-bacterium-specific probe EUB338, Streptococcus-specific probe STR405 and Actinomyces naeslundii-specific probe ACT476. Yellow-green, blue and red represent streptococci, A. naeslundii and other bacteria, respectively. A. XY section in the basal layer of a 24-h biofilm showing spider-like colonies of A. naeslundii in a streptococcus-dominated biofilm. Bar = 25 µm. B. XZ section of 48-h biofilm. Note the preferential location of A. naeslundii in the inner part of the biofilm next to the surface (bottom of the image). Image width = 200 µm. Images from Dige I. Initial dental biofilm formation studied by confocal laser scanning microscopy and fluorescence in situ hybridization. Faculty of Health Sciences, Aarhus University. PhD dissertation (2008). (TIF) [file pone.0025299.s003.tif]

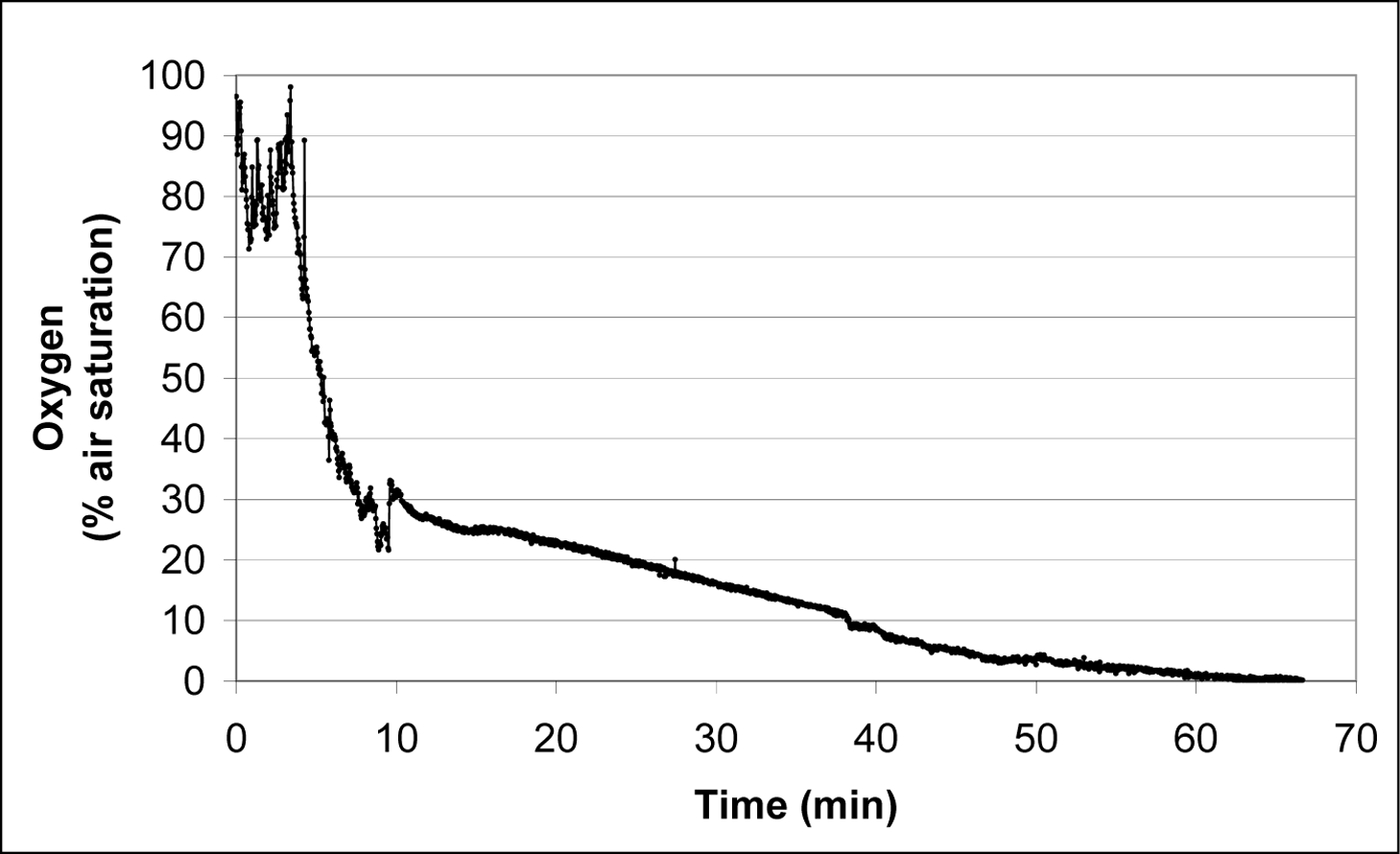

Supplement: Figure S4 — Oxygen concentration in the outport of the flow channel. During flow of sterile saliva containing 10% (w/v) glucose, oxygen concentration in the effluent saliva was close to air saturation. When flow was stopped (t = 4 min), the oxygen concentration decreased until anoxic conditions were reached. (TIF) [file pone.0025299.s004.tif]

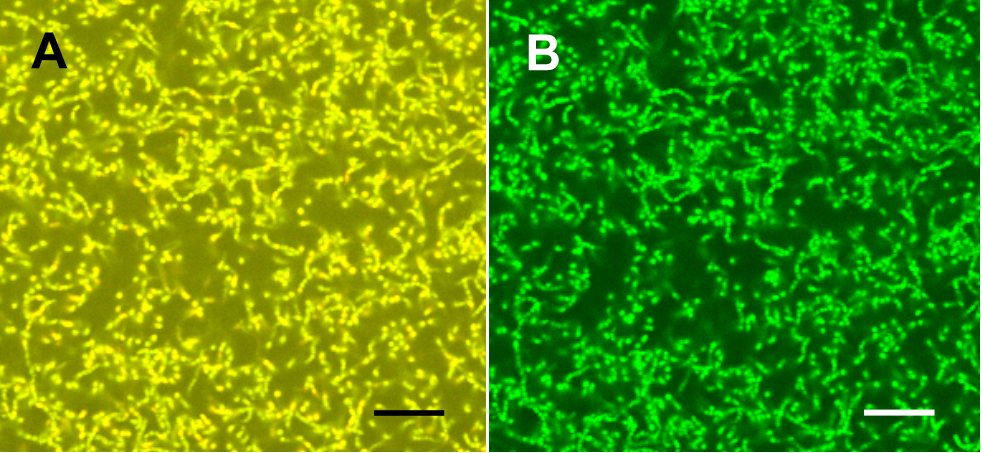

Supplement: Figure S5 — Staining of biofilms with C-SNARF-4 and SYTO9. Both pictures show identical fields of view. Biofilm was first stained with C-SNARF-4 (A), then with SYTO9 (B). All cells detected with SYTO9 were equally visualized with C-SNARF-4. Bars = 20 µm. (TIF) [file pone.0025299.s005.tif]

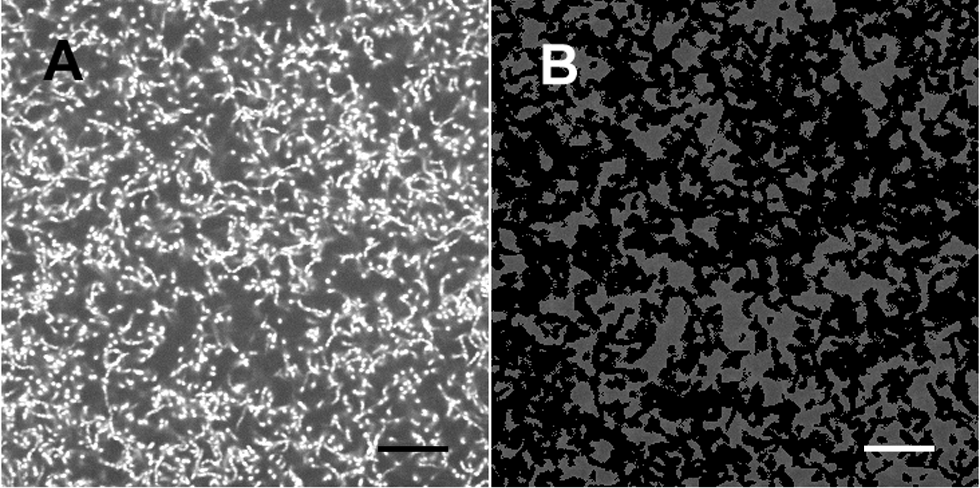

Supplement: Figure S6 — Removal of cells stained with C-SNARF-4 from biofilm images A. Biofilm stained with C-SNARF-4. B. The program daime was employed to remove all cells from the pH data pictures in order to exclusively determine extracellular pH in the biofilm. Bars = 20 µm. (TIF) [file pone.0025299.s006.tif]

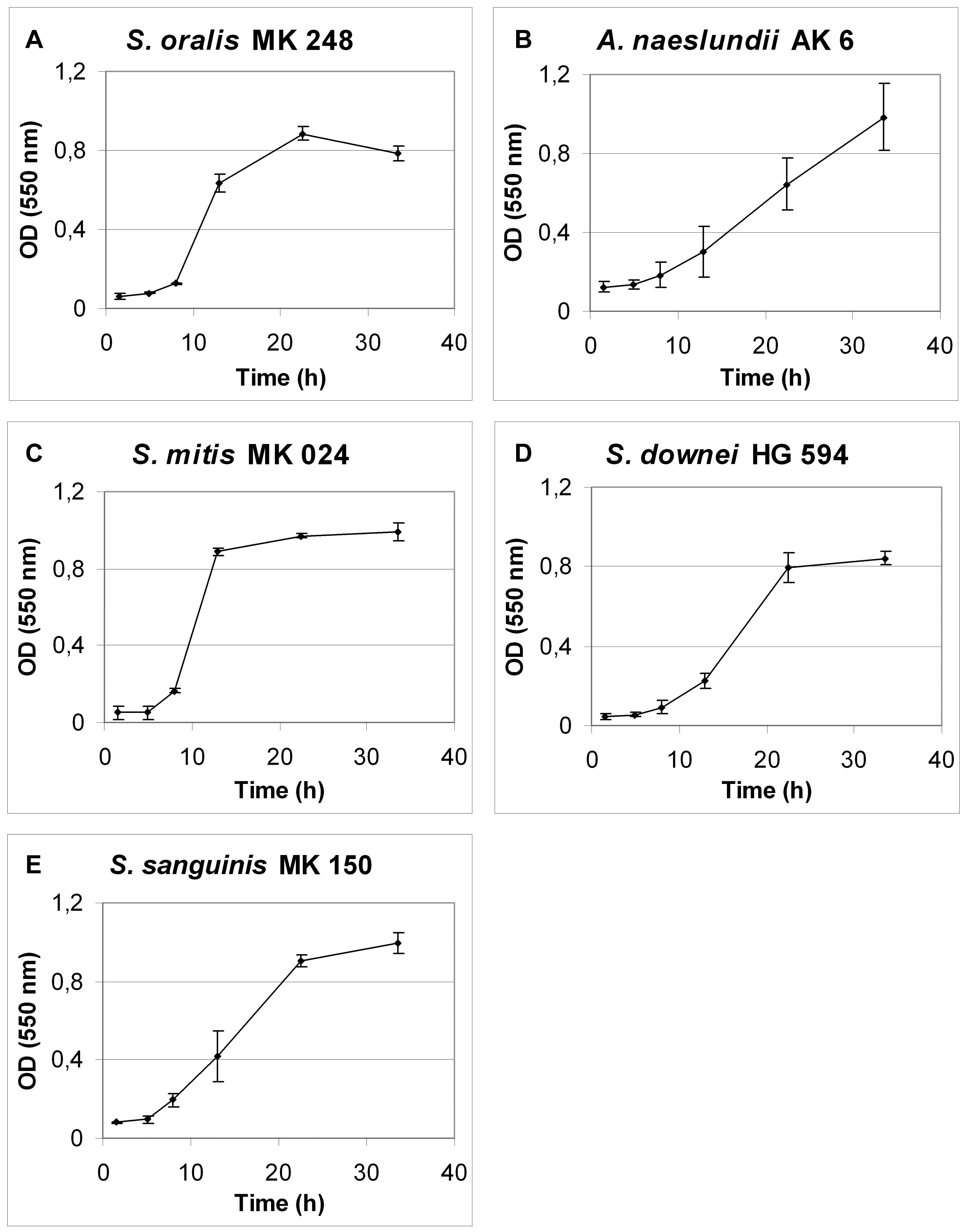

Supplement: Figure S7 — Growth of the bacterial strains in planktonic culture. A. S. oralis SK248. B. A. naeslundii AK6. C. S. mitis SK24. D. S. downei HG594. E. S. sanguinis SK150. Bacteria were grown aerobically in Todd Hewitt Broth at 35°C. Experiments were performed in triplicate. Error bars indicate standard deviations. (TIF) [file pone.0025299.s007.tif]

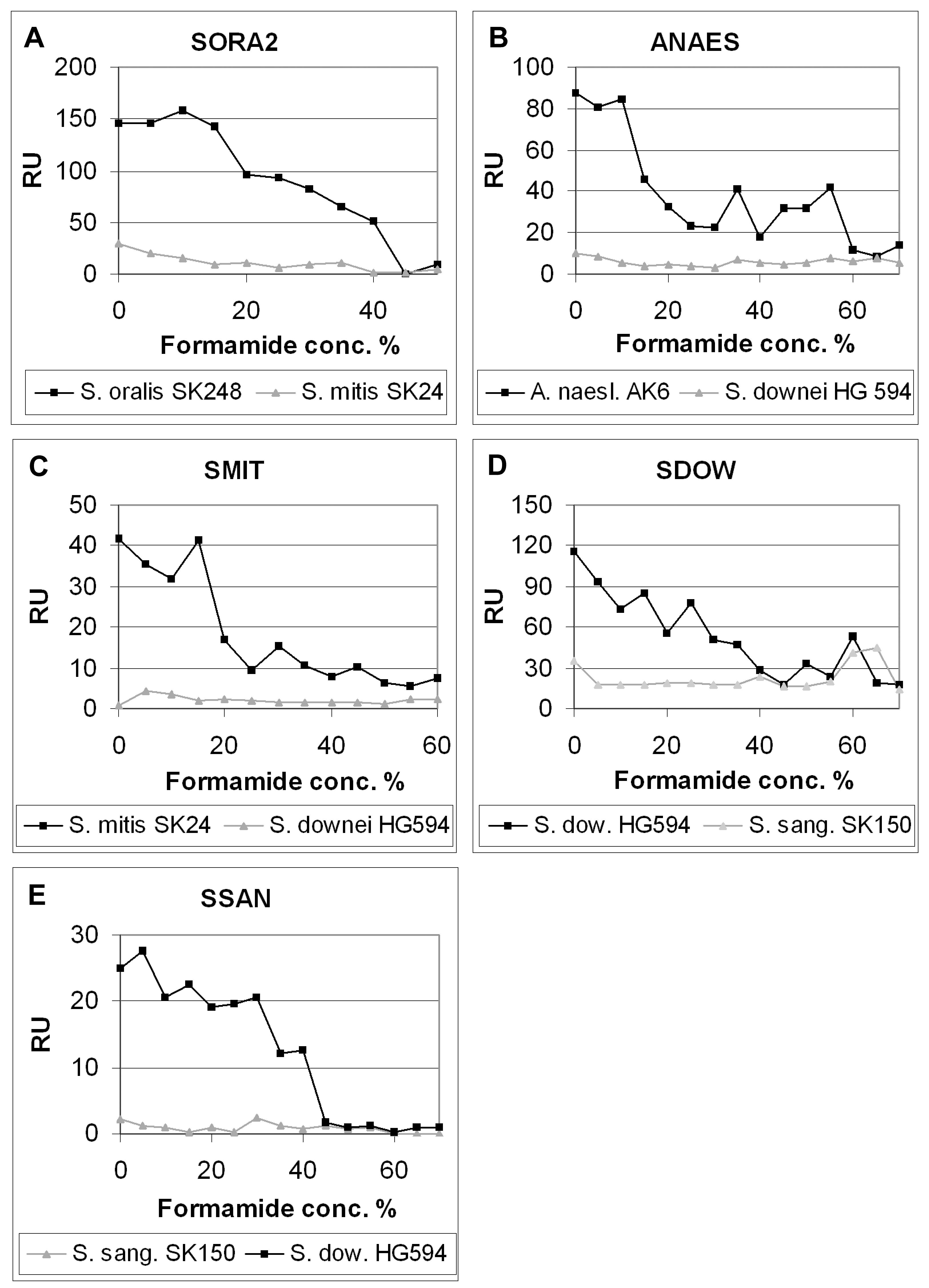

Supplement: Figure S8 — Optimization of FISH conditions for the designed oligonucleotide probes. Probes were hybridized to fixed cells of the positive and negative controls, using varying formamide concentrations in the hybridization buffer. Images with fixed exposure time were acquired, and the signal intensities were measured and given as RU = relative fluorescent units. A. Probe SORA2+helper probe SORA2H. B. Probe ANAES+helper probes ANAESH1 and ANAESH2. C. Probe SMIT+helper probes SMITH1 and SMITH2. D. Probe SDOW+helper probe SDOWH. E. Probe SSAN+helper probe SSANH. Even at low concentrations of formamide the signal from the negative controls was weak. All subsequent FISH experiments were performed at 10% formamide. (TIF) [file pone.0025299.s008.tif]

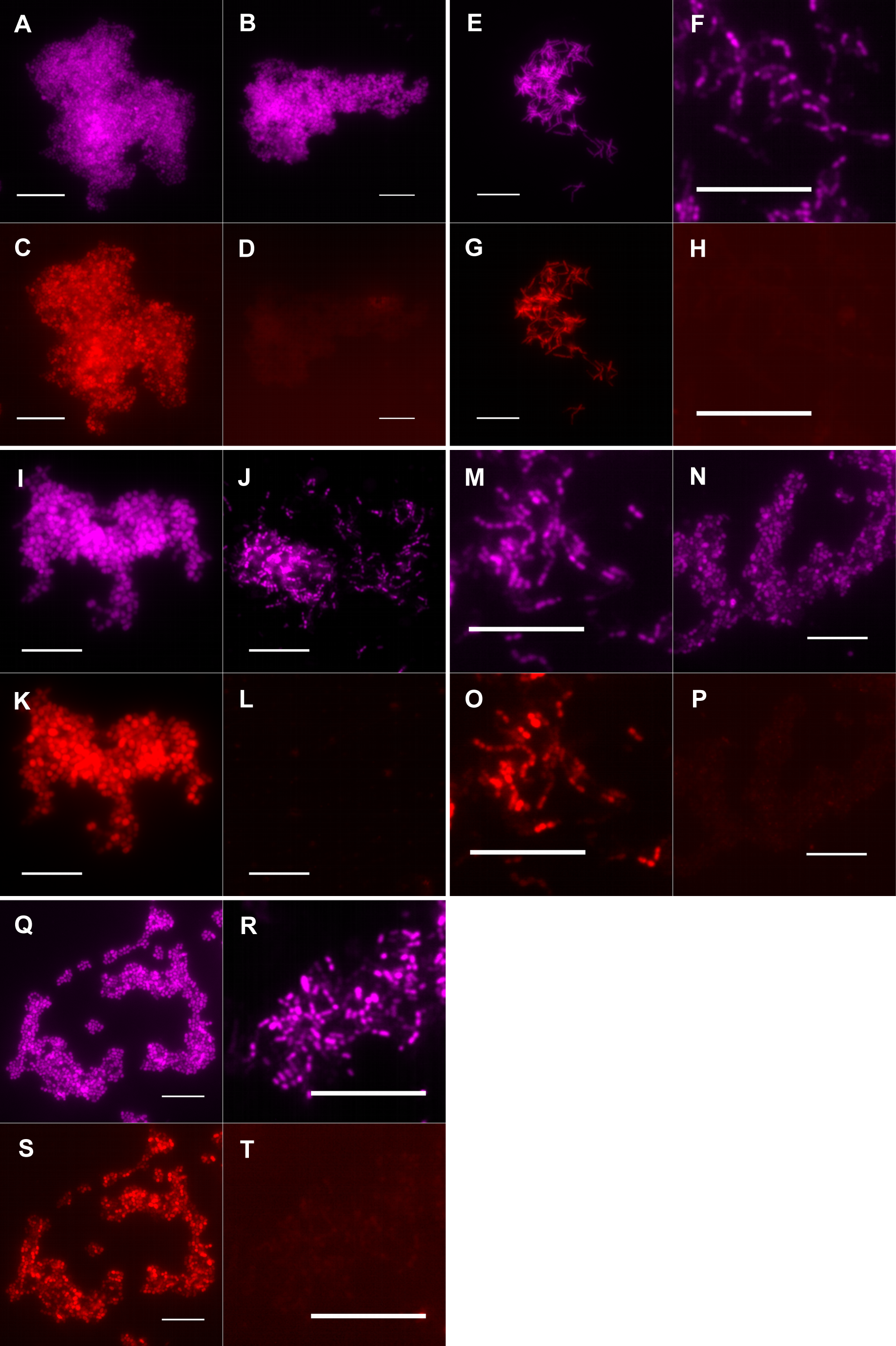

Supplement: Figure S9 — Specificity of FISH probes. Fixed cells of positive and negative control organisms were submitted to FISH with EUB338 (magenta) and the species-specific probes SORA2, ANAES, SMIT, SDOW or SSAN (red). Positive controls S. oralis SK248, A. naeslundii AK6, S. mitis SK24, S. downei HG594 and S. sanguinis SK150 were detected with EUB338 (A, E, I, M, Q) and with the respective specific probes SORA2 (C), ANAES (G), SMIT (K), SDOW (O) and SSAN (S). Negative controls were detected with EUB338 (B, F, J, N, R), but not with the respective specific probes (D, H, L, P, T). Bars = 10 µm. (TIF) [file pone.0025299.s009.tif]

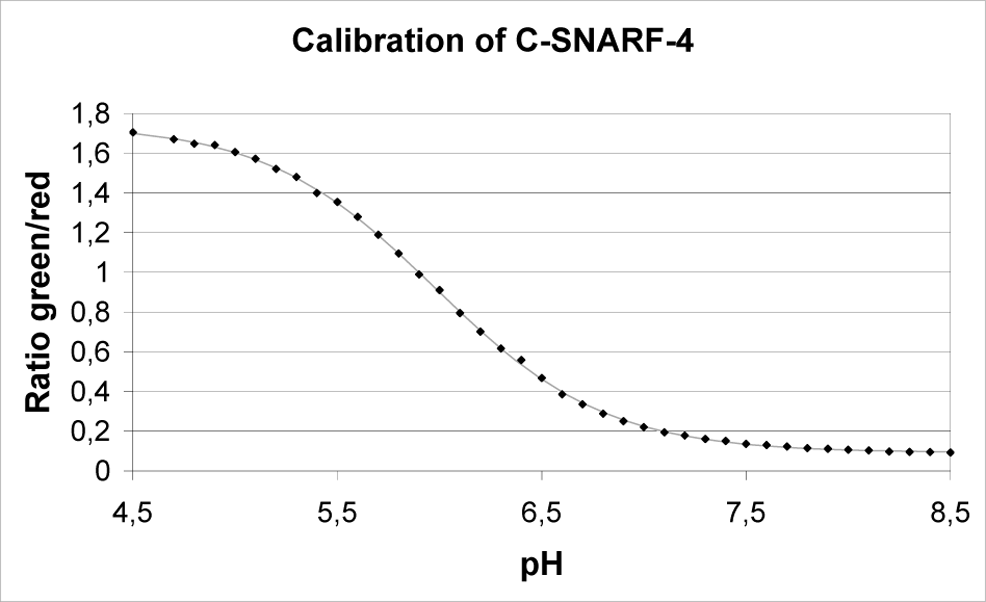

Supplement: Figure S10 — Calibration of C-SNARF-4. Black diamonds show green/red-ratios of C-SNARF-4 fluorescent emissions at different pH values, as determined in HEPES buffer. A sigmoid function was fitted to the ratios and used as calibration curve for the biofilm experiments. (TIF) [file pone.0025299.s010.tif]
