# Supplementary material for: pH Landscapes in a Novel Five-Species Model of Early Dental Biofilm
Source: PLoS One. 2011 Sep 23;6(9):e25299. doi: 10.1371/journal.pone.0025299 (PMC3179500; doi:10.1371/journal.pone.0025299)
Supplement: Table S1 — Sequences of primers used for PCR amplification and sequencing. (DOC) [file pone.0025299.s012.doc]

**Table S1. Sequences of primers for PRC and sequencing.**

| **Primer name** | **Target group** | **Primer sequence** | **Reference** |
| --- | --- | --- | --- |
| 8F | Bacteria; 16S rRNA gene | 5’-AGAGTTTGATYMTGGCTCAG-3’ | [1] |
| EUB 26F | Bacteria; 16S rRNA gene | 5’-AGAGTTTGATCCTGGCTCA-3’ | [2] |
| EUB 518F | Bacteria; 16S rRNA gene | 5’-CCAGCAGCCGCGGTAAT-3’ | [3] |
| Univ 907F | Universal; 16S rRNA gene | 5’-AAACTYAAAGGAATTGACGG-3’ | [4] |
| Univ 907R | Universal; 16S rRNA gene | 5’-CCGTCAATTCCTTTRAGTTT-3’ | [4] |
| Univ 1390R | Universal; 16S rRNA gene | 5’-GACGGGCGGTGTGTACAA-3’ | [5] |
| 1492R | Universal; 16S rRNA gene | 5’-GGYTACCTTGTTACGACTT-3’ | [6] |

**References**

1. Juretschko S, Timmermann G, Schmid M, Schleifer KH, Pommerening-Roser A, et al. (1998) Combined molecular and conventional analyses of nitrifying bacterium diversity in activated sludge: Nitrosococcus mobilis and Nitrospira-like bacteria as dominant populations. Appl Environ Microbiol 64: 3042-3051.

2. Hicks RE, Amann RI, Stahl DA (1992) Dual staining of natural bacterioplankton with 4',6-diamidino-2-phenylindole and fluorescent oligonucleotide probes targeting kingdom-level 16S rRNA sequences. Appl Environ Microbiol 58: 2158-2163.

3. Muyzer G, de Waal EC, Uitterlinden AG (1993) Profiling of complex microbial populations by denaturing gradient gel electrophoresis analysis of polymerase chain reaction-amplified genes coding for 16S rRNA. Appl Environ Microbiol 59: 695-700.

4. Lane DJ, Pace B, Olsen GJ, Stahl DA, Sogin ML, et al. (1985) Rapid determination of 16S ribosomal RNA sequences for phylogenetic analyses. Proc Natl Acad Sci U S A 82: 6955-6959.

5. Zheng D, Alm EW, Stahl DA, Raskin L (1996) Characterization of universal small-subunit rRNA hybridization probes for quantitative molecular microbial ecology studies. Appl Environ Microbiol 62: 4504-4513.

6. Loy A, Lehner A, Lee N, Adamczyk J, Meier H, et al. (2002) Oligonucleotide microarray for 16S rRNA gene-based detection of all recognized lineages of sulfate-reducing prokaryotes in the environment. Appl Environ Microbiol 68: 5064-5081.
