# Supplementary material for: pH Landscapes in a Novel Five-Species Model of Early Dental Biofilm
Source: PLoS One. 2011 Sep 23;6(9):e25299. doi: 10.1371/journal.pone.0025299 (PMC3179500; doi:10.1371/journal.pone.0025299)
Supplement: Table S2 — Sequences of newly designed oligonucleotide probes. (DOC) [file pone.0025299.s013.doc]

**Table S2. Sequences of newly designed oligonucleotide probes.**

| **Probe name** | **Detected organism** | **Probe sequence** |
| --- | --- | --- |
| SORA2 | *Streptococcus oralis* SK248 | 5’-GCACCAAGCTTCAGCGTTCTA-3’ |
| SORA2H | Helper probe *S. oralis* SK248 | 5’-CGTTCGCAACTCATCCGCTCG-3’ |
| ANAES | *Actinomyces naeslundii* AK6 | 5’-GGCGCAATCTTTCCCAGGCCCAC-3’ |
| ANAESH1 | Helper probe *A. naeslundii* AK6 | 5’-CCACCAAAAACACCAAAA-3’ |
| ANAESH2 | Helper probe *A. naeslundii* AK6 | 5’-GCGACAGACCCAGAATAT-3’ |
| SMIT | *Streptococcus mitis* SK24 | 5’-TTAAGCAAATGTCATGCAACATC-3’ |
| SMITH1 | Helper probe *S. mitis* SK24 | 5’-TAGTGATGCAATTGCACC-3’ |
| SMITH2 | Helper probe *S. mitis* SK24 | 5’- CTATTATGCGGTATTAGC-3’ |
| SDOW | *Streptococcus downei* HG594 | 5’-AAGCTCCGGTGATATCAATG-3’ |
| SDOWH | Helper probe *S. downei* HG594 | 5’-CTCATTAATATCGGTGGA-3’ |
| SSAN | *Streptococcus sanguinis* SK150 | 5’- TACGGTATAAACTGTGCGTCCTA-3’ |
| SSANH | Helper probe *S. sanguinis* SK150 | 5’-CAGTCTATGGTGTAGCAA-3’ |
